# Response: Commentary: Memory CD8^+^ T Cells Colocalize with IL-7^+^ Stromal Cells in Bone Marrow and Rest in Terms of Proliferation and Transcription

**DOI:** 10.3389/fimmu.2016.00329

**Published:** 2016-08-31

**Authors:** Özen Sercan-Alp, Andreas Radbruch

**Affiliations:** ^1^Department of Cell Biology, German Rheumatism Research Center, Leibniz Institute, Berlin, Germany

**Keywords:** CD8 T cells, bone marrow, memory, Ki-67, homeostatic proliferation, BrdU, DNA content

In her commentary to our paper (1), Francesca Di Rosa very nicely summarizes past and recent findings in the field of bone marrow (BM)-resident memory CD8 T cells (2) and discusses the lifestyle of memory CD8 T cells in the BM, as circulating cells temporarily staying and proliferating in the BM. Here, in our response, we argue that the available evidence strongly suggests that the vast majority (>90%) of the memory CD8 T cells in BM are resting in terms of proliferation.

We have recently analyzed proliferation of memory CD8 T cells in murine and human BM, murine spleen, and human blood, according to expression of Ki-67, incorporation of Bromodeoxyuridine (BrdU), and DNA content analysis by propidium iodide (PI) staining. Ki-67 is a protein reported to be expressed by cells that are in any stage of the cell cycle, from G1 to M phases, but not in the G0 phase (3). In the memory phase of an immune response, on average, more than 94% of the Ag-specific memory CD8 T cells in murine BM did not express Ki-67 (1) indicating that only a few percent (less than 6%) of the cells are proliferating, if at all. Indeed, staining of the cells for their DNA content with PI revealed that less than 1% of the cells were actively proliferating, i.e., in the S/G2/M phases of cell cycle at any given time point. Similar results for Ki-67 expression of memory CD8 cells in murine BM have been published in the meantime by Geerman and colleagues (4). For memory CD8 cells from human BM and blood, we had described before that, on average, less than 2% of BM or 5% of blood memory CD8 T cells expressed Ki-67 (5). We would like to emphasize that staining for Ki-67 and for DNA content are non-invasive methods, in all likelihood, reflecting the situation *in vivo*.

In previous analyses, the groups of Rafi Ahmed and Francesca Di Rosa had used BrdU and carboxyfluorescein succinimidyl ester (CFSE) to analyze proliferation of BM memory CD8 T cells *in vivo* (6–8). BrdU was fed to mice in drinking water or injected intravenously. After 1 day of BrdU injection, about 7% of the BM memory CD8 T cells proliferated incorporating BrdU into their DNA (6). When the mice were fed with BrdU in drinking water for a longer time period such as 14 days, about 50% of the memory phenotype CD8 T cells became BrdU^+^ (8). Extrapolation suggested a complete turnover of the memory CD8 T cell pool of BM within 1–2 months (8, 9). When we noticed the apparent discrepancy between our Ki-67 and PI stainings and the previously published BrdU-incorporation data, we combined all three approaches. We fed mice with BrdU in drinking water and then stained their memory CD8 T cells for BrdU and Ki-67 or analyzed their DNA content. After 3 days of BrdU feeding, about 60% of the memory phenotype CD8 T cells of BM had incorporated BrdU. Ki-67 staining revealed that almost all of the BrdU^+^ cells, hence 60% of all memory CD8 T cells, also became Ki-67^+^ as opposed to less than 6% in mice not fed with BrdU. Furthermore, the frequency of cells in S/G2/M increased to 5.4%, as compared to 0.4% in mice, which did not receive BrdU in drinking water (1). This result shows that BrdU can induce proliferation of resting memory CD8 T cell of the BM. By comparing BrdU incorporation and CFSE dilution of adoptively transferred CFSE-labeled cells, Parretta and her colleagues showed that CD8 T cell proliferation that is assessed by the loss of CFSE staining was comparable between BrdU-treated and untreated mice (8). However, it should be noted that mice receiving CFSE-labeled CD8 memory T cells were also injected with Polyinosinic:polycytidylic acid (poly:IC), activating the MyD88 pathway. The induction of proliferation of memory CD8 T cells by BrdU is MyD88 dependent, since MyD88 deficient mice do not show Ki-67 upregulation upon BrdU feeding (unpublished data). Thus, in this particular, highly invasive experiment, both poly I:C and BrdU could have induced proliferation. We agree with Francesca Di Rosa that it is difficult, if not impossible, to standardize the uptake of BrdU of individual mice, if the BrdU is provided in the drinking water. Nonetheless, BrdU is clearly no reliable marker for proliferation and results warrant confirmation by alternative (non-invasive) measures.

In summary, recent data from our group and the group of Nolte, using non-invasive measures of proliferation, strongly suggest that proliferation of CD8 memory T cells in the memory phase of an immune response is very low, if it occurs at all (1, 4, 5). What is the nature of the very few memory CD8 T cells, which are proliferating in steady state, as detected by non-invasive analyses? Could they be cross-reactive to self-antigens? Analyzing the entire pool of memory CD8 T cells is more complicated due to the presence of recently activated cells. An indication for this is that, in human BM and blood, some of the Ki-67^+^ memory T cells have downregulated the expression of CD127, the receptor for interleukin 7, indicating their recent activation (5). Nevertheless, by far, the vast majority of memory T cells are resting in terms of proliferation. A fundamental question comes up, namely, whether (homeostatic) proliferation does play any role in the maintenance of CD8 memory T cells, CD4 memory T cells (10), and memory B cells.

## Author Contributions

ÖS-A and AR wrote the text and approved the final submission.

## Conflict of Interest Statement

The authors declare that the research was conducted in the absence of any commercial or financial relationships that could be construed as a potential conflict of interest.
